# Supplementary figures and images for: Multivalent Interactions Drive the Toxoplasma AC9:AC10:ERK7 Complex To Concentrate ERK7 in the Apical Cap
Source: mBio. 2022 Feb 8;13(1):e02864-21. doi: 10.1128/mbio.02864-21 (PMC8822341; doi:10.1128/mbio.02864-21)

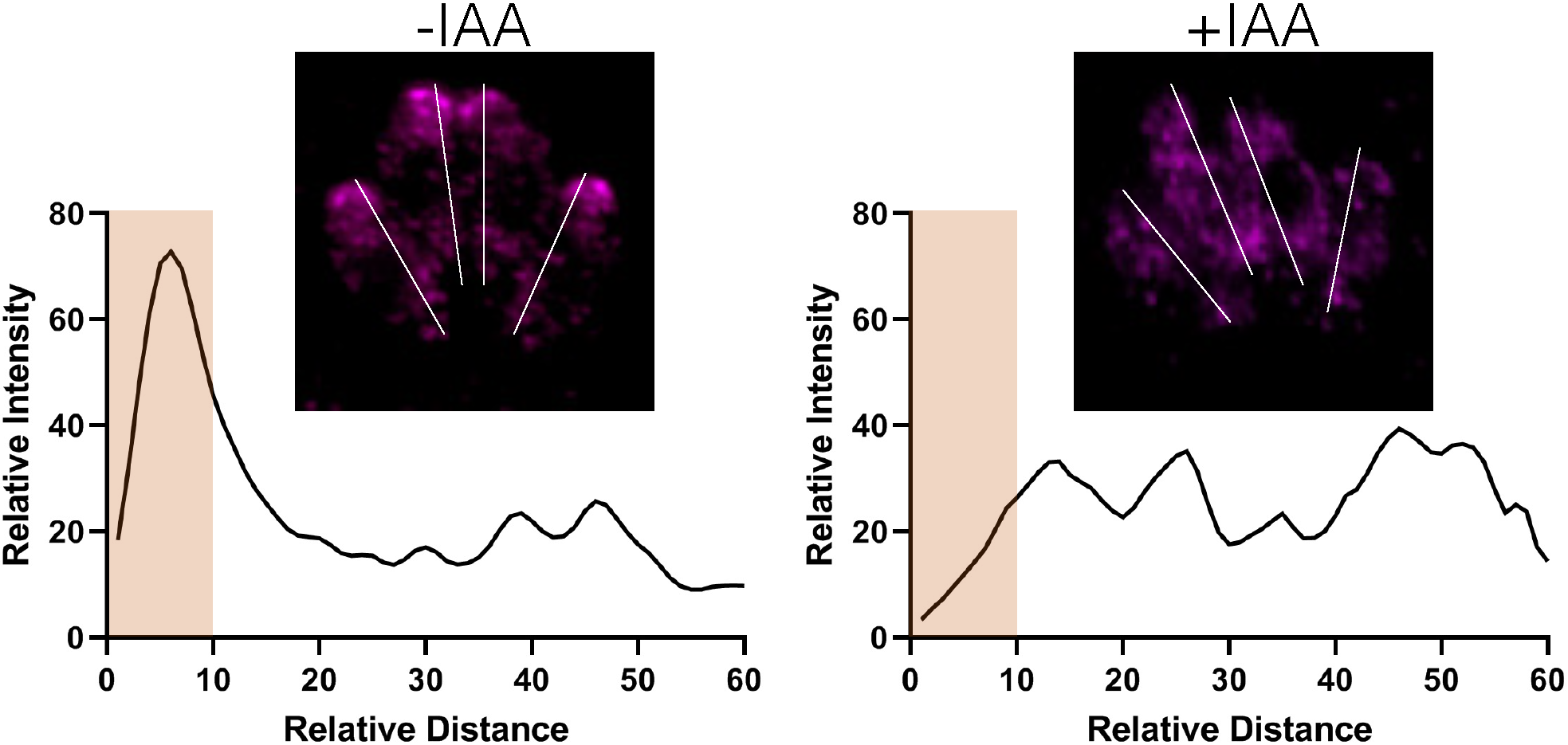

Supplement: FIG S2 [file mbio.02864-21-sf002.tif]

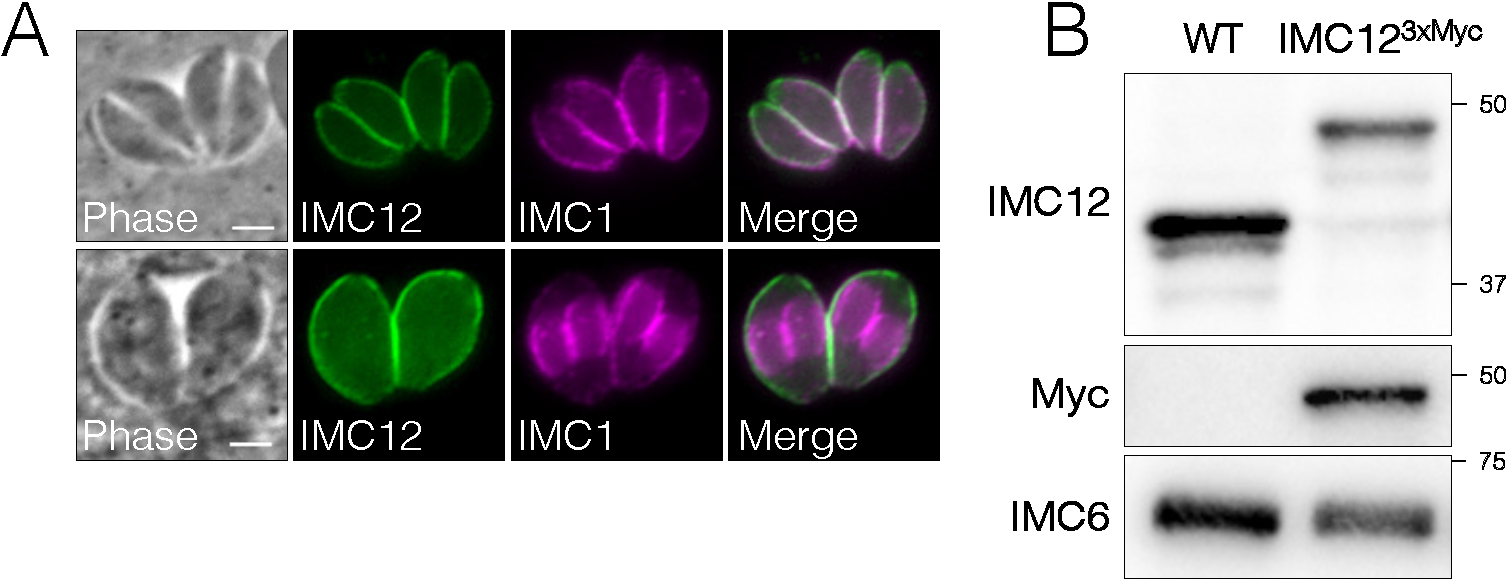

Supplement: FIG S1 [file mbio.02864-21-sf001.tif]

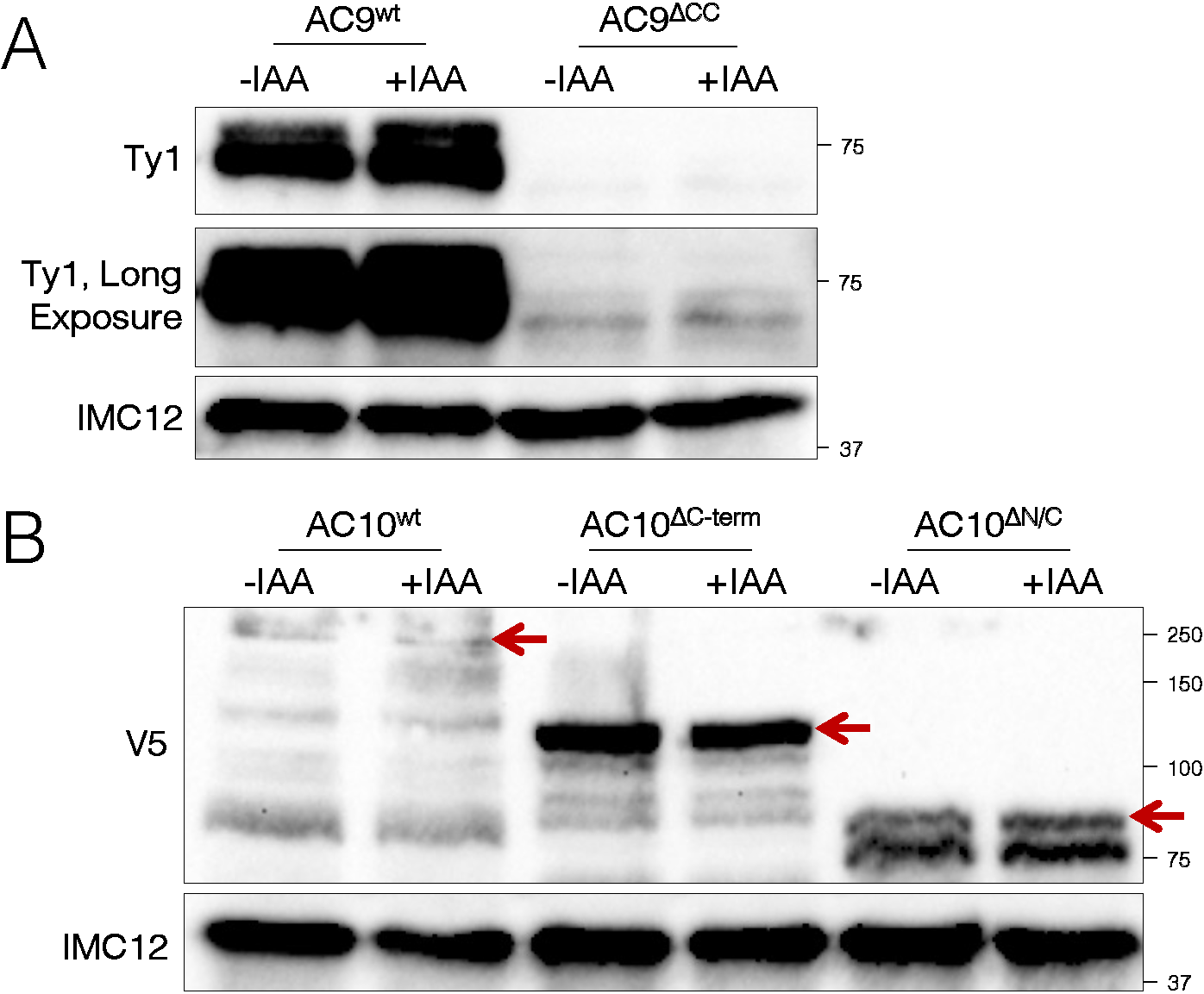

Supplement: FIG S3 [file mbio.02864-21-sf003.tif]

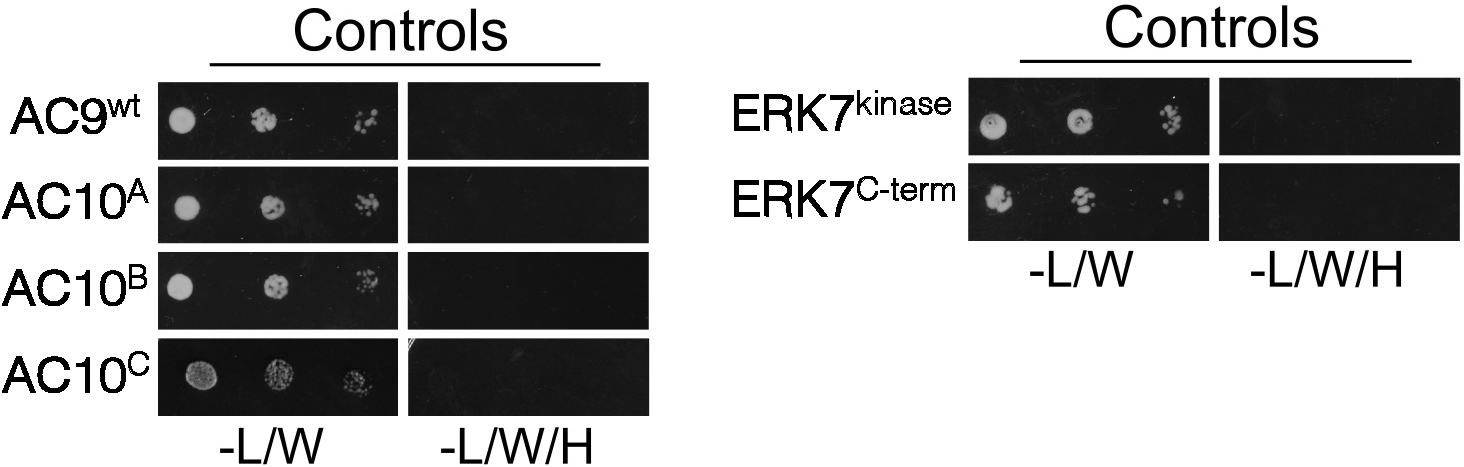

Supplement: FIG S4 [file mbio.02864-21-sf004.tif]
