# Supplementary material for: Multivalent Interactions Drive the Toxoplasma AC9:AC10:ERK7 Complex To Concentrate ERK7 in the Apical Cap
Source: mBio. 2022 Feb 8;13(1):e02864-21. doi: 10.1128/mbio.02864-21 (PMC8822341; doi:10.1128/mbio.02864-21)
Supplement: TABLE S1 [file mbio.02864-21-st001.pdf]

Supplemental Table 1: Oligonucleotides used in this study.

Purpose	Name	Description	Sequence 5'-3'
**AC10 (TGGT1_292950) tagging**	P1	AC10 gRNA-tagging sense	AAGTTgTAAGGGTGCAAGAGTTGATGG
	P2	AC10 gRNA-tagging antisense	AAAACCATCAACTCTTGCACCCTTAcA
	P3	AC10 5' HDR template	GCTGAAGACTCGCCTGATAAAGCTCACGAAGGCAAACACGGGAAGTGGAGGACGGGAATT
	P4	AC10 3' HDR template	AGTCCACTGCTGCTCCTCGAGCAGTCTGGGAGATTTGGCGACGGCCAGTGAATTGTAATA
**AC9 (TGGT1_246950) tagging**	P5	AC9 gRNA-tagging sense	AAGTTATGTGTTCCTCAAATGTCAGG
	P6	AC9 gRNA-tagging antisense	AAAACCTGACATTTGAGGAACACATA
	P7	AC9 5' HDR template	GTCGGGGAACCGCGAACCGAGTGAATATCCGCAGGGAATGGGAAGTGGAGGACGGGAATTC
	P8	AC9 3' HDR template	CTCGCAGCGTGTCGCACTGAACTCTTGTGCGGAGAGAGCGACGGCCAGTGAATTGTAATA
**ERK7 (TGGT1_233010) tagging**	P9	ERK7 gRNA-tagging sense	AAGTTGCAAAAGCAAAGATTCAGACG
	P10	ERK7 gRNA-tagging antisense	AAAACGTCTGAATCTTTGCTTTTGCA
	P11	ERK7 5' HDR template	TTCTCTTTTTTTTCAGTCTGCGTCCAAGACATACAACAGCGGAAGTGGAGGACGGGAATT
	P12	ERK7 3' HDR template	GCTTTCTCCACCTTCGCTTTCGGTGGAAGTCTTGCGAGCGACGGCCAGTGAATTGTAATA
**AC9 CC deletion construct**	P13	AC9 CCdel mut fwd	AAGGAGGCGTTTGCACGCAG
	P14	AC9 CCdel mut rev	GGCTGCTGCAGCCGGGTC
**AC10 promoter and full length complementation**	P15	pUPRTKO vector fwd	GCGGCCGCCTACCCGTAC
	P16	pUPRTKO vector rev	ATGCATATGCGATGTCGAACCCTCG
	P17	AC10pro-coding fwd	gttcgacatcgcatatgcatCCCCACTCGTTCCTCGATG
	P18	AC10pro-coding rev	tcgtacgggtaggcggccgcTCTCGCCTTTAATTGCAGTACG
**AC10 CC1 deletion construct**	P19	AC10CC1 Q5del fwd	CTGACGCATGCAGTGGAC
	P20	AC10CC1 Q5del rev	GTCACCGCAGGTGGCATC
**AC10 AC9-BD deletion construct**	P21	AC10-AC9bd Q5del fwd	AAACTAGACGCTGAAGAC
	P22	AC10-AC9bd Q5del rev	GTTATCTTCTGCACTGCC
**AC10 CC2 deletion construct**	P23	AC10CC2 Q5del fwd	GAAAGTACAGGAAGTCGTG
	P24	AC10CC2 Q5del rev	ACAGACATTCGATCGTTG
**AC10 N-term deletion construct**	P25	AC10N-term Q5del fwd	GGAGACACGCGTCCGCAT
	P26	AC10N-term Q5del rev	CATCGCAACTTCCTCCTTTTCG
**AC10 C-term deletion construct**	P27	AC10C-term Q5del fwd	GCGGCCGCCGGCAAACCT
	P28	AC10C-term Q5del rev	GGACTGAGCAGGTAGAACTGAAAGGGATAGAGG
**AC10 N/C deletion construct**	P29	pUPRTKO-AC10wt-vector-fwd	GCGGCCGCCGGCAAACCT
	P30	pUPRTKO-AC10wt-vector-rev	CATCGCAACTTCCTCCTTTTCGCAAAAAGATGTGTTCTTTC
	P31	AC10N-Cdel fwd	aaaaggaggaagttgcgatgCTTACGCGTCTAGATTTG
	P32	AC10N-Cdel rev	ataggtttgccggcggccgcGGACTGAGCAGGTAGAAC
**IMC12 pET28 construct for antibody production**	P32	IMC12-pET28 fwd	TAATAACATTGGAAGTGGATAAC
	P33	IMC12-pET28 rev	TGCATTGGATTGGAAGTAC
	P34	IMC12-coding fwd	tgtacttccaatccaatgcaGCAACCGAGTTCGTCGTTC
	P35	IMC12-coding rev	atccacttccaatgttattaCTGGGGCATGGAGTCGAC
**Yeast-2-hybrid**	P36	f pB27* ga/PhQC [58]	TAAGGGCCACTGGGGCCCC
	P37	r pB27 ga/PhQC [59]	TCCGGCCCCGAATTCCAGC
	P38	f pP6 3p ga/PhQC [57]	CTCGAGTAGCTAGTGTCTAGAG
	P39	r pP6 5p ga/PhQC [57]	ATTCGTGGCCCCTGCGG
	P40	f AC9(D2) pb27-ga [58]	ctggaattcggggccggaGACGTCTCCGGTCGAGGC
	P41	r AC9(M452*) pb27-ga	gggccccagtggcccttaCATTCCCTGCGGATATTCACTCG
	P42	f AC9(P70) blunt [61]	CCGGCTGCAGCAGCCATTC
	P43	r AC9(Q113*) blunt [59]	tcaCTGCTCCTCCAAGCTTTTGAAAG
	P44	r AC9(A157*) blunt [59]	tcaGGCAAAGCCTTGAATGGTTGAGG
	P45	f AC10(V2) pP6-ga [57]	gccgcaggggccacgaat GTGACTGCAGTACCCAATCCTTC
	P46	r AC10(N650*) pP6-ga [57]	ctagacactagctactcgag tca GTTATCTTCTGCACTGCCTTTGAC
	P47	f AC10(E651) pP6-ga [59]	gccgcaggggccacgaatGAAGACAGCACAGAGAGCCAG
	P48	r AC10(T1300*) pP6-ga [59]	ctagacactagctactcgagtcaGGTGGTACGCGTCGCTATTC
	P49	f AC10(S1301) pP6-ga [57]	gccgcaggggccacgaatTCCCCTTTTCAGCGGGGAG
	P50	r AC10(R1979*) pP6-ga [57]	ctagacactagctactcgagTCATCTCGCCTTTAATTGCAGTAC
	P51	f AC10(K684) blunt [59]	AAACTAGACGCTGAAGACCAGAAG
	P52	r AC10(R683) blunt [58]	GCGTGGATCAAGCACCTTGG
	P53	r AC10(S913*) blunt [58/62]	tcaGGACTGAGCAGGTAGAACTG
	P54	f AC10(W914) blunt [59/64]	TGGAATACTACGTCTGTGTCCGC
	P55	r AC10(C780 no*) [57/61]	ACAGACATTCGATCGTTGCTG
	P56	f AC10(E831) [57/61]	GAAAGTACAGGAAGTCGTGCG
	P57	r TgERK7(A358*) blunt [61]	tcaAGCTGTGCGGTGTCGCCG
	P58	f TgERK7(G359) blunt [59]	GGTTCTTCCGGCCGCCACC
